# Regulatory Divergence of Transcript Isoforms in a Mammalian Model System

**DOI:** 10.1371/journal.pone.0137367

**Published:** 2015-09-04

**Authors:** Sarah Leigh-Brown, Angela Goncalves, David Thybert, Klara Stefflova, Stephen Watt, Paul Flicek, Alvis Brazma, John C. Marioni, Duncan T. Odom

**Affiliations:** 1 University of Cambridge, Cancer Research UK - Cambridge Institute, Li Ka Shing Centre, Cambridge, United Kingdom; 2 European Molecular Biology Laboratory, European Bioinformatics Institute, Wellcome Trust Genome Campus, Hinxton, Cambridge, United Kingdom; 3 Wellcome Trust Sanger Institute, Wellcome Trust Genome Campus, Hinxton, Cambridge, United Kingdom; 4 California Institute of Technology, Division of Biology, Pasadena, California, United States of America; University of Chicago, UNITED STATES

## Abstract

Phenotypic differences between species are driven by changes in gene expression and, by extension, by modifications in the regulation of the transcriptome. Investigation of mammalian transcriptome divergence has been restricted to analysis of bulk gene expression levels and gene-internal splicing. Using allele-specific expression analysis in inter-strain hybrids of *Mus musculus*, we determined the contribution of multiple cellular regulatory systems to transcriptome divergence, including: alternative promoter usage, transcription start site selection, cassette exon usage, alternative last exon usage, and alternative polyadenylation site choice. Between mouse strains, a fifth of genes have variations in isoform usage that contribute to transcriptomic changes, half of which alter encoded amino acid sequence. Virtually all divergence in isoform usage altered the post-transcriptional regulatory instructions in gene UTRs. Furthermore, most genes with isoform differences between strains contain changes originating from multiple regulatory systems. This result indicates widespread cross-talk and coordination exists among different regulatory systems. Overall, isoform usage diverges in parallel with and independently to gene expression evolution, and the *cis* and *trans* regulatory contribution to each differs significantly.

## Introduction

Changes in the regulation of gene expression during evolution can cause differences between species in total transcript abundance and/or the proportions of represented isoforms [1, 2]. Many studies have dissected the changes in levels of gene expression, as well as the genetic mechanisms that underlie this divergence [1, 3–5]. The set of isoforms expressed from a gene is as tightly controlled as the gene expression level, both between individuals and between cells from the same tissue [6]. However, the extent to which a gene’s isoform usage changes between closely related mammalian subspecies and the mechanisms that might underlie such changes, have remained unexplored.

Multiple diverse and independent regulatory systems contribute to the set of isoforms expressed from a gene. These contributions impact not only internal splice site choice, but also promoter selection, transcription start site selection, and polyadenylation site selection [7–11]. Isoform usage divergence contributes to organismal evolution by modulating post-transcriptional regulatory sequences embedded within a transcript, as well as changing protein structure [12, 13].

Regulatory systems that control transcript structure involve an interaction between nucleic acid sequences in DNA or RNA (*in cis*) and protein or RNA-based complexes binding to them in a sequence-specific fashion (*in trans)*. Promoter selection and transcription start site selection are regulated by transcription factors and cofactors binding to sequences in gene enhancers and promoters, which control the behavior of the basal transcription machinery through recruitment to a specific site, or alter post-translational modifications on the basal transcription factors [8, 9, 14]. Internal splicing, in contrast, is controlled by the spliceosome, a large ribonucleoprotein complex that assembles through a series of intermediates on sequences in the target intron and flanking exons and catalyzes intron excision [15]. Alternative internal splicing occurs due to differential binding of splice factors such as HnRNP and SR proteins, or by changes in sequence at the 3’ and 5’ exonic splice sites and the intronic branch site [15]. Polyadenylation site selection is poorly understood, despite 50% of human genes containing alternative polyadenylation sites [16], but it is believed to be controlled by recruitment of cleavage factors to sequences in the nascent RNA transcript, which in turn recruit the poly-A polymerase [17, 18].

To date, most studies of isoform expression divergence have focused on internal splice site choice, including exon gain/loss and cassette exon inclusion [7, 12, 13, 19]. In C. elegans, eQTL studies have analyzed internal splicing divergence between strains, and found that *cis*-acting variants predominate [20]. In fruit flies, intercrosses of Drosophila species and subspecies have been used to thoroughly dissect genome-wide the mechanisms underlying divergence of internal splicing [7]. They observed that patterns of alternative splicing have distinct profiles of *cis* and *trans* divergence. For example, intron retention is predominantly driven by *cis*-regulatory changes, whereas exon skipping is equally driven by mutations *in cis* or *in trans*. Some studies in human cells have taken a quantitative trait analytical approach, and identified a number of proximal genetic variants associated with heritable changes in splicing in HapMap lymphoblastoid lines [21–24].

Comparison of splicing across all vertebrate clades revealed that cassette exon expression levels diverge at a significantly higher rate than gene expression levels [12, 25]. Divergence of cassette exon expression and divergence of gene expression appears to be decoupled in vertebrates, displaying independent evolution both between tissues and between species [12, 26]. Between evolutionarily distant mammals, divergence of cassette exon expression levels are driven primarily by *cis*-regulatory changes, as observed in a transchromic mouse stably carrying human chromosome 21 [12]. The degree to which variation of isoform usage is driven by selection or drift is unclear; analysis of exon usage across six primates suggested that a minority of changes in exon usage are functional and under selective pressure [27].

Here, we use a classical genetics approach to dissect transcriptome divergence using inbred mouse strains as a mammalian model species. We have analyzed the divergence of internal splicing, transcription start site selection, polyadenylation site selection, and promoter choice. Our results quantify the mechanisms contributing to evolutionary divergence in transcriptional and post-transcriptional isoform usage, and how they conspire with differential gene expression to generate transcriptional divergence.

## Results

### Isoform usage differences are as widespread as gene expression differences in a single mammalian species

**Fig 1:**
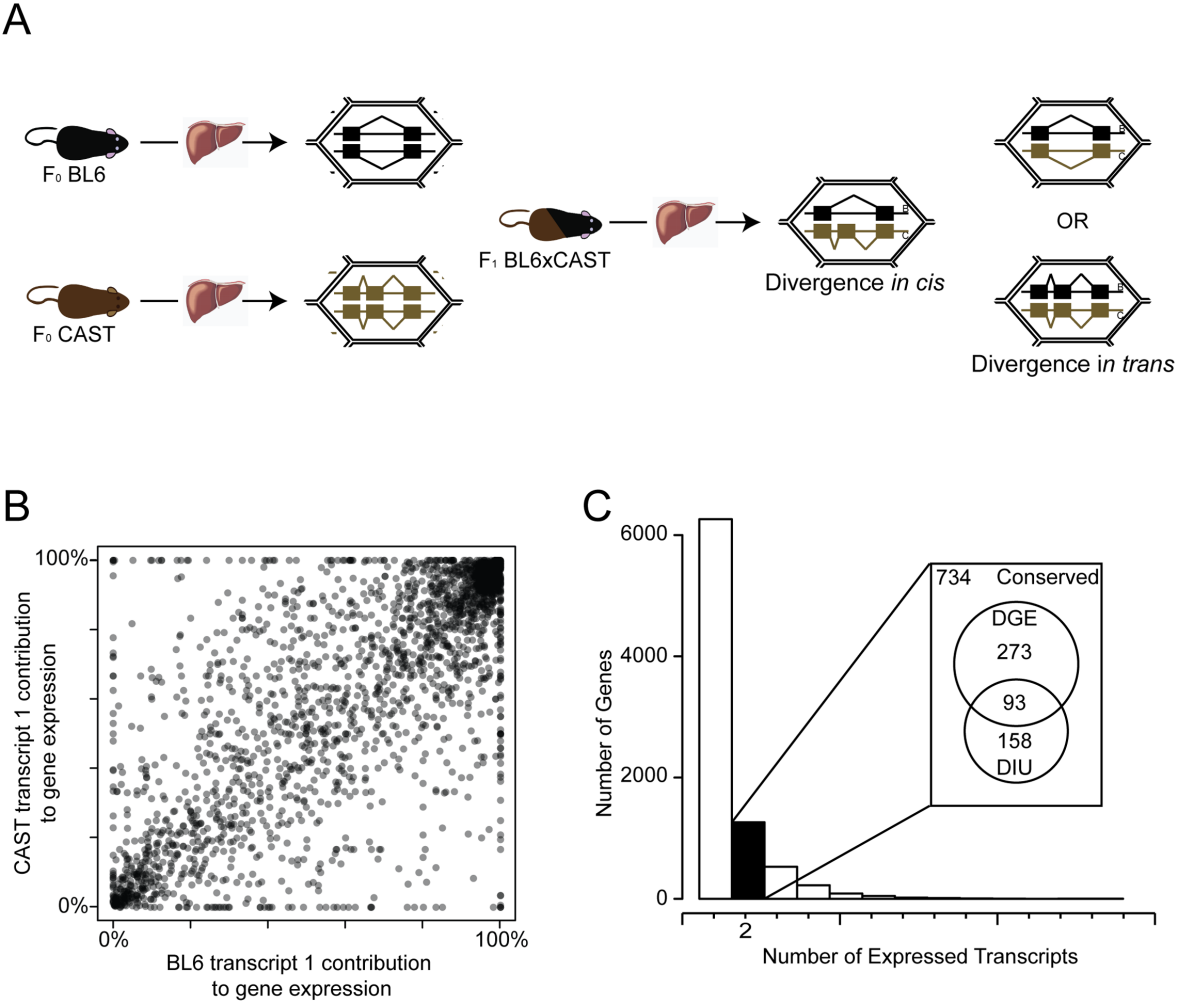
Divergent Isoform Usage (DIU) between closely related mouse subspecies. (**A)** Experiments interrogated DIU by comparing the parental F0 strains, and both directional crosses of F1 mice. Illustrative examples are shown of purely *cis* and *trans* driven divergence of isoforms. (**B)** Divergence of transcript expression between liver transcriptomes of male BL6 and CAST mice. Each point is one gene expressing two transcripts: the x-axis is the proportion of total gene expression in F0 BL6 which is derived from one transcript; the y-axis is the proportion of total gene expression in F0 CAST which arises from the same transcript. (**C)** Histogram of the number of genes (y-axis) binned by the number of expressed transcripts observed in male mouse liver (x-axis). Genes expressing only two transcripts were studied (black bar) to detect divergent isoform usage (DIU). Venn diagram callout shows the overlap of genes expressing exactly two transcripts and levels of Divergent Gene Expression (DGE) in the same sample set [28].

We used genetic crosses of two mouse strains, previously used to study imprinting, gene expression evolution and methylation [28–30]. C57Bl6/J (BL6) is an inbred strain derived from *Mus musculus domesticus*, while CAST/EiJ (CAST) is an inbred strain derived from *Mus musculus castaneus*. Therefore, this system assays regulatory divergence that has arisen during the 500,000 years since these subspecies shared a common ancestor [31, 32]. We previously generated RNAseq libraries from 6 male inbred BL6 and CAST mice—the parental/F0 groups—and 12 hybrid F1 male offspring of BL6 and CAST (Fig 1A) [28]. Here we extend this previous work, which focused on overall gene expression levels, by re-analysing this data to generate transcript expression estimates from each group using MMSEQ (Fig 1A, Methods) [33]. To discriminate between multiple differential isoform usage (DIU) regulatory changes within a gene and also from changes to overall gene expression, we considered a set of genes expressing only two overlapping transcripts in adult mouse liver (2211 genes). This set represents 50% of all genes expressing multiple isoforms, and includes both genes encoding many transcripts of which only two are expressed, and genes for which only two transcripts are annotated, where both are expressed. Restricting analysis to only genes expressing two isoforms gave us the power to precisely characterize the regulatory change necessary to alter the ratio of the two isoforms, therefore isolating specific regulatory change events at each locus. Due to the allele-specific nature of our analysis we further subset this group to genes containing one or more known SNV or indel between BL6 and CAST (set of 1258 genes used for further analysis).

According to Genetrail enrichment analysis, this group was not enriched for any GO term, KEGG, Transfac or Transpath pathway relative to the full set of expressed genes (p < 0.001, not shown), therefore we considered it to be a representative subset. Genes expressing 3, 4 or 5 isoforms were not enriched for any KEGG, Transpath or Transfac pathway, however moderate enrichment was observed for GO categories associated with subcellular location: genes expressing 3 isoforms are enriched for the GO terms cytoplasm (p = 0.0001) and organelle membrane (p = 0.00015), genes expressing 4 isoforms are enriched for mitochondrion (p = 8.1e-5), cytoplasmic part (p = 8.1e-5), and cytoplasm (p = 0.0004), and genes expressing 5 isoforms are enriched for the GO term cofactor binding (p = 0.0006). Relative to all expressed genes, single-isoform genes are enriched in a number of functional GO categories (25 in total of p < 0.001), suggesting that single-isoform genes, which were outside the scope of our analysis, may have specific functional characteristics.

Of this set of 1258 genes, 20% showed expression patterns consistent with a divergence in the contribution of each isoform to total gene expression between species (251 genes) (Fig 1B). Of these 251 genes, 100 have altered protein-coding sequence between the two isoforms (40%), and 13 genes modified the proportion of transcripts subject to nonsense-mediated decay (5%) (S1 Table).

We considered the possibility that isoform usage and differential gene expression were entwined. Recent work describes divergence of gene expression regulation (DGE) in liver using the same RNAseq libraries, so we compared our measurement of DIU to DGE in the same sample set [28]. 37% of the genes with robust DIU between BL6 and CAST also have DGE, which is approximately what would be expected by chance, therefore, DIU and DGE are most likely occurring independently (χ^2^ test, p > 0.1) (Fig 1C, S1 Table). This is consistent with the observation that population-level variability of gene expression and of splicing at a locus are independent [34].

### Most isoform expression differences between BL6 and CAST involve multiple regulatory systems

**Fig 2:**
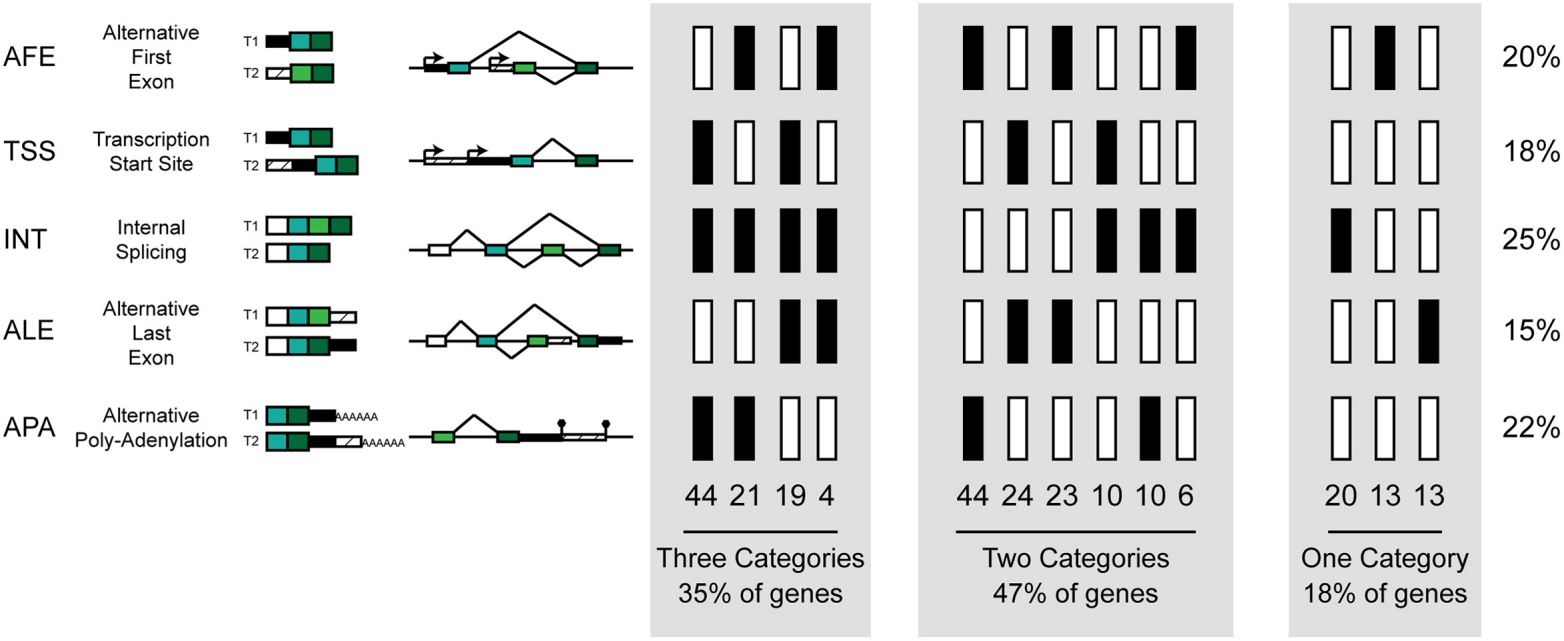
Divergent Isoform Usage of a single gene most often involves mechanistic contributions from multiple regulatory systems. Genes with differential isoform usage were categorized according to the differences in transcript structure between the two expressed isoforms: Alternative First Exon (AFE), Transcription Start Site (TSS), Internal Splicing (INT), Alternative Last Exon (ALE), and Alternative Poly-Adenylation (APA). All five categories of structural change are illustrated on the left, and the number of isoform pairs with each combination of structural differences is shown by columns (black indicates the presence of the structural change, white indicates the absence). For example, a gene expressing two isoforms which differ by both alternative first exon and alternative polyadenylation site usage has both AFE and APA and therefore is counted in the 5^th^ column from left, and in total there were 44 genes like this. The summary statistic at the bottom of each grey panels indicates the number of genes with any combination of 3 structural changes, 2 structural changes or only a single change.

The final structure of a transcribed isoform is the product of multiple regulatory processes, including promoter selection by transcription factors, transcription start site selection by the basal transcriptional machinery, splice site selection by the spliceosome, and poly-adenylation site selection by the poly-A polymerase complex [8–11]. We dissected the contribution of these regulatory systems to DIU in mouse by analysis of structural differences between expressed isoform pairs: (i) differential promoter usage creating alternative first exons [AFE], (ii) alternative transcription start site selection [TSS], (iii+iv) alternative splicing, altering either internal [INT] or last (terminal) exons [ALE], and (v) alternative poly-adenylation [APA] (Fig 2). Genes producing a pair of non-overlapping transcripts were removed for this analysis (5 genes, 0.4%). All five of these mechanistic categories can alter the structure and/or expression of the final protein, and thus its activity, regulation, and/or cellular localization. Further, AFE, TSS, ALE and APA all lead to different 5' or 3' UTR sequence, and so could alter transcript regulation by RNA binding proteins and microRNAs.

The 251 overlapping isoform pairs with DIU between BL6 and CAST contained in total 544 discrete differences in isoform structure, meaning that most isoform expression changes involved multiple regulatory systems (Fig 2, S1 Table). The five categories were present in similar frequency (15%, 18%, 20%, 22%, 25% of isoform structure changes were ALE, TSS, AFE, APA, and INT, respectively) but ALE/APA and AFE/TSS are, by definition, mutually exclusive. The INT category was more frequently observed without any other divergence event than expected by chance (χ^2^ test, p << 0.001). Genes with divergent transcript expression due to internal splicing (INT) were enriched for sequence predicted to encode coiled-coils (Genetrail, p < 0.007) No other category of transcript structural change was enriched with any KEGG, GO, TRANSPATH or TRANSFAC category, or well-known sequence motif (Methods).

### Unlike gene expression divergence, mammalian isoform divergence is often caused by regulatory changes *in trans*

**Fig 3:**
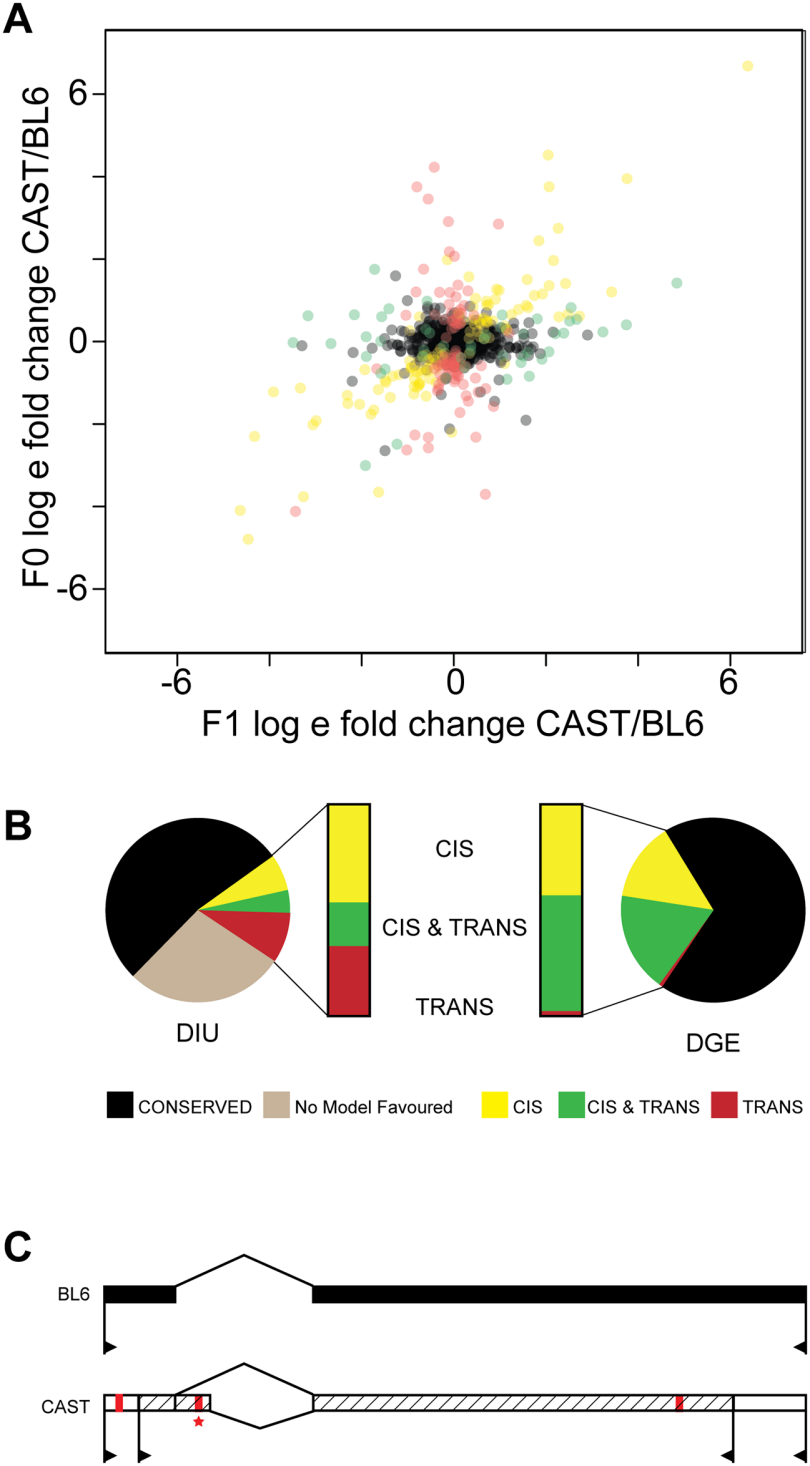
Divergent Isoform Usage is caused equally by regulatory changes *in cis* and *in trans*. Genes were classified according to the mechanism underlying their DIU: conserved, regulatory changes *in cis*, *in trans*, *in cis* & *in trans*, or genes where no model was significantly favored over the rest **(A)** Scatterplot shows each heterozygous gene expressing exactly two isoforms in liver, plotting the mean fold change in the ratio of CAST to BL6 transcript expression in the F0 (F0 BL6 v F0 CAST) against the F1 (BL6 allele in F1 v CAST allele in F1), weighted by the inverse of the estimate variances (**B)** The relative contribution of *cis* and *trans* mechanisms towards transcriptome changes differed significantly between divergent isoform usage (DIU) and divergent gene expression (DGE) in the same sample set [28]. (**C**) Divergent isoform usage is encoded *in cis* to the Commd5 gene. In F1 hybrid offspring, the BL6 allele expresses a single transcript (*Commd5-001*, black) and the CAST allele expresses two transcripts (*Commd5-001*, white, and *Commd5-002*, hatched). Commd5-001 and Commd5-002 utilise different transcription start sites (>), alternative internal splicing, and discrete polyadenylation sites (<). SNV between BL6 and CAST are indicated in red. * Indicates rs32416751, predicted to disrupt the 5’ splice site in Commd5.

The mammalian transcriptome is regulated by interactions between proximal nucleic acid sequences that are genetically linked to the target gene ***in cis***, and sequence-specific binding proteins and RNAs that can diffuse throughout the nucleus and act ***in trans***. As a result, any mutation causing divergent transcript expression is either encoded *in cis* or *in trans* to the target locus. Analysis of allele specific expression in F1 hybrids can determine whether changes occur *in cis* or *in trans* [35]. A regulatory change encoded *in cis* is inherited in an allele-specific fashion; in contrast, a regulatory change encoded *in trans* is mediated by a diffusible element and therefore regulates both alleles equally (see Fig 1A for a hypothetical example). Allele specific isoform usage was measured in twelve biological replicates of the F1 hybrids, and expression estimates were obtained using MMSEQ (Methods) [33]. Of the 1258 loci expressing two overlapping transcripts, isoform usage was divergent in 251 (20%), and isoform usage was conserved in 684 (54%); whereas for 323 genes (25%), neither conservation nor divergence was statistically favored (Fig 3A, Methods). We focused our analysis on the 251 divergent genes (Fig 3B, left hand colored bar). We found that just under half (116, 46%) had regulatory divergence encoded only *in cis*, a third had regulatory changes only *in trans* (89, 35%), and about a fifth had divergence *in cis* and *in trans* acting on the same gene (46, 18%) (S1 Table, S1 Fig).

A comparison with previously published analyses of gene expression divergence generated from the same RNAseq dataset revealed significant regulatory differences underlying gene expression and isoform deployment (Fig 3B) [28]. Most notably, *trans-*regulatory changes are observed frequently in DIU (35%), whereas DGE is almost never driven by changes only *in trans* (2%) [28]. Changes in gene expression levels between BL6 and CAST are predominantly driven by compound effects of independent mutations *in cis* and *in trans* (55%); in contrast, isoform changes are caused by compound effects no more frequently than expected by chance (18%) (χ^2^ test, p > 0.1) [28]. Only 9% of loci had the same regulatory change in both DIU and DGE, indicating that gene expression and isoform usage are most likely driven by independent and functionally orthogonal regulatory mutations (χ^2^ test, p > 0.1) (Fig 3B, S1 Table). To confirm that our results were not biased by the inclusion of only genes expressing precisely two isoforms, we performed a similar analysis on the major isoform arising from genes expressing precisely 3, 4, 5, or 6 or more isoforms (S4 Fig). Despite this approach being less powerful than the method used for 2 isoforms, it demonstrates that genes expressing many isoforms have a very similar proportion of changes *in cis* and *in trans*.

Mutations in *trans* often affect multiple loci; we therefore tested whether the *trans* regulatory changes underlying isoform divergence were downstream of specific functional regulatory pathways. No significant associations were observed between KEGG, GO, TRANSFAC or TRASPATH functional categories and mechanisms of divergence (Genetrail, p > 0.01). We asked whether specific isoform structural changes are enriched for regulatory mutations *in cis* or *in trans*, relative to the conserved group. A locus with DIU encoded *in trans* is less likely to contain differences in the last exon (ALE) than a locus with DIU encoded *in cis* (Fisher’s Exact Test, p = 0.008) (Table 1). We then searched for potentially causative genetic variants near genes with transcripts that show *cis*-encoded regulatory mutations using Ensembl’s Variant Effect Predictor [36]. For instance, the gene *Commd5* expresses a single isoform in BL6 (*Commd5*-001), and an additional, second isoform is also highly expressed in CAST (*Commd5*-002) (Fig 3C). Analysis of allele-specific expression in the F1 mice indicates that the underlying regulatory mutation is encoded *in cis*. The CAST-specific transcript employs a different 5’ splice site in its 5’ UTR (INT) than the shared transcript, and we identified a variant in the *Mus castaneus* genome that disrupts the consensus splicing motif precisely at this exon-exon junction (rs32416751) (Fig 3C). This mutation plausibly explains the underlying mechanism of divergence for this locus. More globally, approximately 51% of genes carrying a mutation *in cis* have genetic variants in the region of the splice junction, like that identified for *Commd5*.

**Table 1:** Categories of transcript regulation are enriched for classes of regulatory divergence.

	*Conserved*	*in cis*	*in trans*	*in cis and in trans*
**AFE**	38%	38%	51%	48%
**ALE**	37%	45%	25%	26%
**TSS**	45%	41%	36%	39%
**APA**	44%	39%	56%	52%
**INT**	54%	48%	53%	67%

**Fig 4:**
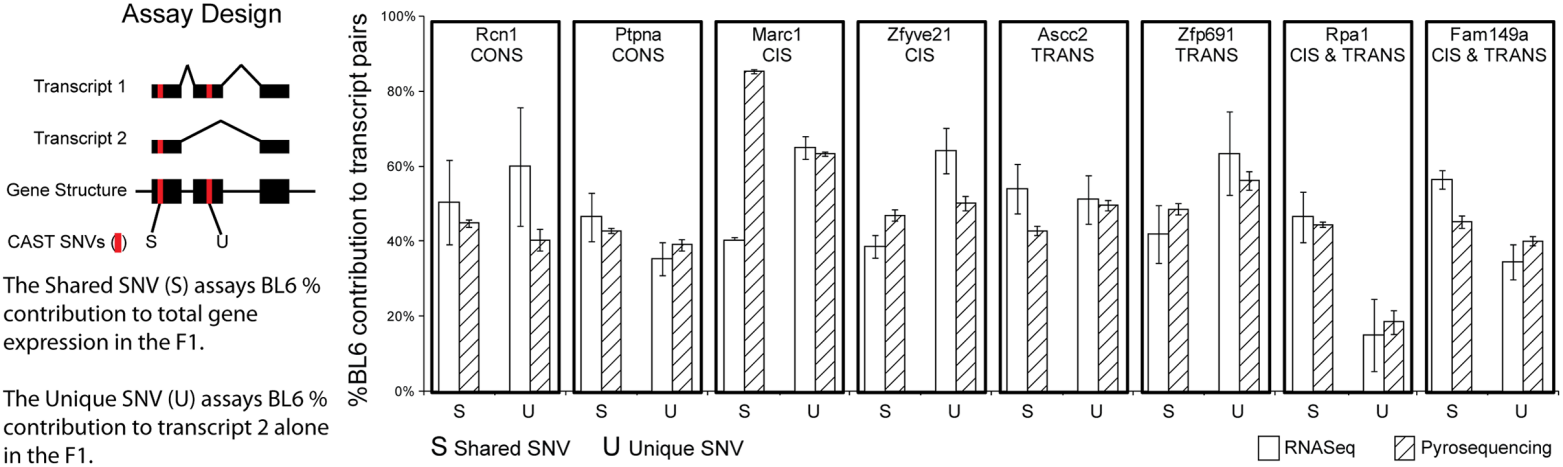
Allele-specific isoform divergence was validated pyrosequencing in the F1. The contribution of the BL6 allele to gene and transcript expression in the F1 hybrids was validated by site-specific pyrosequencing. For each of the eight genes tested, two independent SNVs were assayed: one SNV measured the contribution of the BL6 allele to total gene expression (S), the other assayed the BL6 contribution to one of the two expressed transcripts (U). The pyrosequencing results measuring BL6 contribution to total gene expression (S) and to transcript 2 only (U) should both be in agreement with the RNASeq/MMSeq Expression Estimates. Good agreement was observed for 7 of the 8 genes (Rcn1, Ptpna, Zfyve21, Ascc2, Zfp691, Rpa1, Fam149a).

We validated our results using pyrosequencing for each class of regulatory divergence. The contribution of the BL6 and CAST alleles to overall transcription in F1 mice was confirmed with a pair of allelic expression assays. The first assay used an SNV in an exon shared by both expressed isoforms to determine the proportion of overall gene expression attributable to the BL6 allele. The second assay interrogated an SNV located in an exon found only in one of the two expressed isoforms, to evaluate the contribution of BL6 to that specific isoform (Methods, S2 Table). The pyrosequencing validation results were then compared to MMSEQ expression estimates. Of the 8 loci tested by a pyrosequencing assay pair, 7 demonstrated strong consistency with RNAseq results (Rcn1, Ptpna, Zfyve21, Ascc2, Zfp691, Rpa1, Fam149a) and 1 did not (Marc1). Overall the correlation between RNASeq and Pyrosequencing results was 0.50 (Spearman’s correlation, p < 0.05) (Fig 4).

### Splicing factor sequence and expression level can influence divergence of isoform usage

The expression level of splicing factors can influence isoform usage between different tissues [2], so we asked whether changes in expression levels of the splicing machinery between subspecies could drive the observed evolution of transcript usage. Of 73 well-characterized splice regulators expressed in the liver of CAST and BL6, seven have detectable gene expression divergence (S3 Table) [28]. We performed motif enrichment analysis for a set of 94 splice factors, of which 5 are known to be differentially expressed between BL6 and CAST. We predicted that this analysis would reveal a set of downstream targets whose divergence was encoded, at least in part, *in trans*. To our surprise, we found enrichment for the motif of the well-known splicing factors Hnrnpc and Rbm47 in a set of exons with regulatory divergence encoded *in cis* (adj. p < 0.001, fold change 1.1x and 1.3x, respectively, S4 Table) [37]. Hnrnpc and Rbm47 are both expressed highly in mouse liver, falling in the top 25% of genes ranked by expression level; furthermore Hnrnpc is differentially expressed between BL6 and CAST (overexpressed in BL6, log_2_ fold change -0.489, adj. p < 0.032)[28]. There are numerous differences in the transcribed sequence of the RBM47 gene in CAST relative to BL6: 17 SNVs and 2 INDELS throughout the gene result in transcript sequence changes to all five known Rbm47 isoforms.

## Discussion

### Our work extends the current understanding of splicing evolution in vertebrates

We used allele specific expression analysis in an F1 hybrid system of the mouse strains C57BL/6J (BL6) and CAST/EiJ (CAST) to identify regulatory divergence in isoform usage (DIU) over the time since *Mus musculus domesticus* and *Mus musculus castaneus* last shared a common ancestor, approximately 500,000 years ago [31, 32]. To date, many studies of transcript evolution in fruit flies and mammals have focused on internal splicing regulation [1, 3–5]; our analysis of how mammals control transcript structure and expression extends these studies by including transcription start site selection, promoter choice, and polyadenylation site selection. Within vertebrates, gene-internal splicing diverges more rapidly than gene expression, and the complexity of alternative splicing appears greater in the primate clade than in other vertebrate lineages [12].

For this work we relied on allele specific isoform level expression estimates, which we inferred from RNA-sequencing. Since it is currently not feasible to obtain the full-length sequences of RNA transcripts in a high-throughput manner, in this work we chose to perform expression quantification by aligning RNA-sequencing reads to annotated cDNA sequences and using a probabilistic model to deconvolve expression levels.

Almost one in five assayed isoform pairs had robust evidence of isoform expression divergence between these strains of mice. Remarkably, isoform expression changes in 84% of the divergent loci were caused by changes in multiple underlying regulatory systems. In other words, there were multiple, simultaneous differences in usage of upstream UTRs, downstream UTRs, transcription start sites, and/or their internal exon-exon splicing junctions. Statistically, the co-occurrence of these differences is highly unlikely to be the result of random chance. Three possibilities exist to explain this co-occurrence of regulatory divergences: (i) a single mutation in an upstream regulatory pathway affecting multiple systems; (ii) enrichment of independent regulatory mutations at specific loci due to natural selection; or (iii) an underlying coordination of apparently independent gene control mechanisms, diverging simultaneously by selection or neutral drift. The first possibility is unlikely because a pleiotropic upstream regulator would have been detected in our functional enrichment analysis. The second is unlikely because the recent divergence time of these two mouse strains precludes extensive selective pressure, particularly in light of the high conservation of liver gene expression. The third possibility is therefore the most likely; indeed, co-transcriptional splicing is a well-understood precedent that coordinates the spliceosome and polymerase machineries. Our data reveal that this coordination extends to other aspects of transcript structure such as polyadenylation and transcription start site selection [38, 39]. Also consistent with our results, there have been a small number of reports of splicing factors controlling other aspects of transcript structure such as polyadenylation [9, 40–43]. It is likely that many aspects of transcript structure are codetermined during transcription.

Internal splicing is found in the absence of any other structural change more often than expected by chance. Besides internal splicing, our data revealed that 83% of diverged transcripts between closely related mice have different 5’ UTR sequences (AFE and TSS), and 81% have different 3’ UTR sequences (ALE and APA). Divergence by internal splicing is strongly associated with the presence of the coiled-coil sequence motif. Since there is no functional category associated with this structural change, the reason for this correlation is not clear. This motif is commonly associated with complex regulatory systems involving homo- and hetero-dimerization with multiple partners, including the famous regulators c-fos and c-jun [44].

Our data shows further that divergence of the untranslated regions at both the beginning and end of genes is more common than internal splicing changes. The widespread differences found in the 3’ and 5’ UTRs may substantially alter transcript stability and microRNA recognition sequences. It has long been known that protein coding sequences are rarely altered between closely and even distantly related mammals [45, 46]; however, regulatory alterations that modify the transcriptome’s dynamics have been largely unexplored.

### Differences between inter-species and intra-species regulatory divergence in mammals

Our results reveal that divergence *in cis* and divergence *in trans* both play important roles in transcript usage change within a mammalian species. Previous work in *Drosophila* and *C*. *elegans* has revealed that internal splicing divergence between species of invertebrates is driven predominantly by regulatory changes *in cis* [7, 20]. Similarly in evolutionarily distant mammals, analysis of species-specific internal splicing in a mouse carrying a human chromosome demonstrated a significant enrichment for *cis*-regulatory changes [12]. Thus divergence of internal splicing between many complex eukaryotic species appears to be driven by a similar pattern of regulatory mutation. In contrast, we reveal here a role for *trans*-regulatory variants in internal splicing divergence within a mammalian species. Our results suggest that a transition from a combination of *cis*- and *trans*- regulatory variants to predominantly *cis*-regulatory variants may occur around speciation, which is consistent with observed patterns of intra- and inter-species gene expression divergence in yeast, fruit flies, and mammals [4, 28, 47]. Extending our analysis to subspecies pairs with different divergence times in mammals could reveal the underlying dynamics of these evolutionary mechanisms.

RNA motifs of both the known splice regulator Hnrnpc and Rbm47 were significantly enriched in the exonic sequence of genes that have proximally encoded divergence of isoform expression (S4 Table). The observed differential expression of Hnrnpc between these two strains of mice suggests that the regulatory changes observed *in cis* downstream of Hnrnpc could be compensatory in nature, despite the surprising finding that no evidence of *trans*-encoded divergence was identified. Interestingly, the RBM47 gene in CAST contains more than 20 changes in sequence when compared to the sequence in BL6, many of which contribute to all 5 known isoforms expressed of this gene. It is likely therefore that both differential expression and sequence divergence in splicing factors are altering the transcriptome during the short divergence time between these two subspecies of Mus musculus.

Note added in proof: Since the original submission of this paper, a similar work focusing on fibroblasts from the same mouse strains, as well as a re-analysis of reference 28 has appeared in the literature [48].

### Synopsis

Differential usage of isoforms is prevalent in mammals even following a short divergence time, collectively restructuring both the coding and the noncoding transcriptome. Our results have implications for our understanding of speciation and regulatory divergence, which to date has focused primarily on total gene expression levels. The genome-wide structural differences of transcripts, including polyadenylation, start site selection, internal splicing, and promoter choice, are processes that evolve independently from gene expression levels. Our study has revealed in a single integrated analysis how the interplay of multiple, independent regulatory mechanisms, which include transcriptional regulation, spliceosome function, and polyadenylation [49], are coordinated to shape the transcriptome and its divergence over a short timescale in mammals.

## Methods

### Animal housing and handling

All mice used in this work were housed and handled in accordance with the Animals (Scientific Procedures) Act 1986. Mice were sacrificed by cervical dislocation, and all work was approved by AWERB, Animal Welfare and Ethical Review Body. AWERB is the full name of the ethics committee that approved this study. Full details of animal housing and handling are described in Goncalves et al. [28].

### Sample Preparation and Sequencing

This manuscript uses the same RNAseq data as in Goncalves et al., and full details of animal housing and handling, nucleic acid extraction and QC, library preparation and sequencing are described in that manuscript [28]. Briefly, six biological replicate samples were used for each mouse genotype: C57BL/6J (BL6), CAST/EiJ (CAST), CAST/EiJxC57BL/6 (CASTxBL6), and C57BL/6JxCAST/EiJ (BL6xCAST). Strand-specific RNA-Seq libraries were prepared using the method of Parkhomchuk et al, and sequenced at single end 36bp on an illumina GAIIx in the Genomics Core facility of the CRUK Cambridge Institute.

### Pyrosequencing

Genes were randomly selected from each category of expression divergence (cis, trans, cis & trans, conserved), following exclusion of genes expressing more than two isoforms and genes where isoforms showed evidence of parent-of-origin biased expression. Single nucleotide variants (SNVs) were identified in each isoform pair such that one SNV was shared between both isoforms and the other was unique to a single isoform. Allele specific quantification analysis was performed on both SNVs independently by pyrosequencing in biological triplicate on cDNA from liver of each mouse genotype (BL6, CAST, BL6xCAST and CASTxBL6). Complementary DNA was generated from total RNA using the Superscript II double-stranded cDNA kit (Invitrogen). Primer design, primer validation testing, and pyrosequencing assays were performed by Barts and the London Genome Centre. Sequences targeted by assays are given in S2 Table.

### Estimating isoform expression levels

Isoform levels were estimated as described in Goncalves, Leigh-Brown et al. 2012 [28]. Briefly, reads were aligned to a reference transcriptome using Bowtie [50]. Reads from the F0 mice were mapped to either the BL6 or CAST reference transcriptome (Ensembl 70), as appropriate. For the F1 mice, we aligned reads to a reference containing both the BL6 and the CAST transcriptomes. To ensure that we have an accurate annotation for CAST we performed de novo isoform reconstruction using the Scripture and Augustus tools [51, 52]. The confident set of novel exons completely detected by both tools comprised 99 and 150 novel exons in BL6 and CAST, respectively, giving us confidence that the Ensembl annotation is also an acceptable representation of the CAST transcriptome. Subsequently, MMSEQ was used to estimate isoform expression levels and, in the case of the F1 samples, to estimate allele-specific isoform expression levels. Isoforms were deemed expressed when the expression estimate was above a threshold *t* in at least 4 replicates of either the BL6 F0 samples or the CAST F0 samples and in at least 4 replicates of the F1 samples. The threshold, *t*, was determined as the minimum expression of isoforms with at least 10 unique reads.

Given the complexity of splicing, deconvolving isoform level estimates is difficult. The power to obtain a reliable estimate for an isoform depends on the number of reads mapping uniquely to it, which in turn depends on the length of the region that is unique to the isoform and on the number of reads overlapping it. This power should be reflected by the Monte Carlo standard errors (MCSEs) provided by MMSEQ (S2 Fig). Using simulated data we observed that the correlation between the measurements improved when using isoform subsets under differing MCSE thresholds (S3 Fig). Data was simulated as described in [28]. Briefly, we sampled reads from two F0 libraries (one BL6 and one CAST) and combined them to generate a simulated F1. We then compared, for each transcript, the expression estimate for the BL6 allele in the simulated F1 hybrid with the expression estimate of the same transcript for the F0 sample.

### Classifying divergence of isoform expression

To classify genes according to their mode of expression divergence we defined four models (conserved, cis, trans and cis&trans) as described in Turro et al. 2013 (Section 4.2). We compared the conserved model with each of the three other models and, assuming a prior probability of 0.25 that any of the four models is true, we calculated the joint posterior probability of the models. Genes with a posterior probability greater than 0.5 for any of the models were classified accordingly, while genes for which the data did not favor any model strongly were not considered further. Note that for the sake of stringency we only selected a model if it was more likely than all the other models put together.

### Classifying structural differences in isoform pairs

To determine structure differences between each pair of isoforms we wrote our own R scripts. These scripts first compare whether the isoforms have overlapping first exons. If the first exons overlap and have different start sites, the isoforms are said to have an alternative TSS. If the first exons do not overlap the isoforms are said to have an AFE. ALE and APA are defined in a similar way. Detection of internal splice sites includes checking for exons that are present in only one of the isoforms and also internal splice junctions whose start or end sites differ between the isoforms.

### Over- and Under-Enrichment Analysis

Enrichment analysis was performed using the Genetrail Over-/Under-representation analysis tool by Mus musculus Ensembl gene ID. Enrichment analysis of regulatory divergence classes compared the list of genes in each class (cis, trans, cis&trans, conserved) with the list of all genes expressing 2 transcripts and containing a SNV between BL6 and CAST, and included KEGG-pathway analysis, Transpath-pathway analysis, Transfac analysis, and Gene Ontology analysis. Enrichment analysis of categories of transcript change compared the list of genes containing each structural variant (INT, ALE, AFE, APA, TSS) with the list of all genes expressing 2 transcripts and containing an SNV between BL6 and CAST, and included KEGG-pathway analysis, Transpath-pathway analysis, Transfac analysis, and Gene Ontology analysis; as well as testing for enrichment of chromosomal locations, Pfam domains, miRNA targets, ELR motifs, RGD motifs, and Coiled-coil motifs. In all cases p-value adjustment was performed using FDR adjustment with a significance threshold of 0.01 and a minimum number of genes of 5.

### Motif Enrichment Analysis

To assess whether genes with DIU were enriched for motifs of known RNA binding proteins, we obtained a list of 118 experimentally determined motifs for factors encoded by known mouse genes [37]. Subsequently, we performed pairwise enrichment of the exonic sequences of genes in the different regulatory categories (e.g. cis vs trans, cis vs cons, etc...). Significance was determined using a hypergeometric test using the HOMER software package [53] and multiple testing correction by FDR.

## Supporting Information

S1 Fig Model Selection. The difference between the posterior probability of the best model and the posterior probability of the second best model is plotted for genes with a posterior probability greater than 0.5 for any of the models.
(TIF)

S2 Fig Expression level and MCSEs. The expression levels of all transcripts in one of the F0 libraries are plotted against the respective Monte Carlo standard errors. The MCSEs are related to the expression level of the gene and to the number of reads uniquely mapping to the transcript.
(TIF)

S3 Fig Validation in an *in silico* F1 dataset. To quantify our ability to estimate allele specific isoform expression we created an artificial F1 library as described in Goncalves and Leigh-Brown et al. and compared the original expression levels to the deconvolved ones (Goncalves, Leigh-Brown et al. 2012). (A) When comparing the expression in the F0s to the allelic expression in the F1s without sub-setting by the MCSEs we found a very good agreement between the two (Pearson correlation > = 0.89). However, expression at the isoform level is less well estimated than at the gene level (Pearson correlation > = 0.97). (B) When sub setting the set of isoforms to only the ones under a MCSE threshold t (t in {t_1,t_5} corresponding to the maximum SE among isoforms with {1,5} unique reads) the agreement improves (Pearson correlation > = 0.95).
(TIF)

S4 Fig Extrapolation to genes with many >2 isoforms. To confirm that inclusion of only genes expressing precisely 2 isoforms does not introduce a bias to the analysis, we selected the major isoform in genes expressing 3, 4, 5, or 6 (or more) isoforms and characterized them according to whether their expression in the F1 was consistent with conservation (black) or with divergence in cis (yellow), in trans (red), in cis and in trans (green). Grey indicates loci where no single model was statistically favored over the others. X-axis: number of isoforms expressed from locus, Y-axis: proportion of genes where major-isoform is most likely to have diverged due to each regulatory mechanism.
(TIF)

S1 Table Table of all genes expressing 2 isoforms in adult mouse liver. (XLSX)

S2 Table Table of regions targeted for pyrosequencing validation. (XLSX)

S3 Table Table of known regulators of splicing assessed for differential expression in mouse liver. (XLSX)

S4 Table Enrichment of known splice regulator motifs in genes with divergent isoform usage. (XLSX)
