# Supplementary figures and images for: Regulatory Divergence of Transcript Isoforms in a Mammalian Model System
Source: PLoS One. 2015 Sep 4;10(9):e0137367. doi: 10.1371/journal.pone.0137367 (PMC4560434; doi:10.1371/journal.pone.0137367)

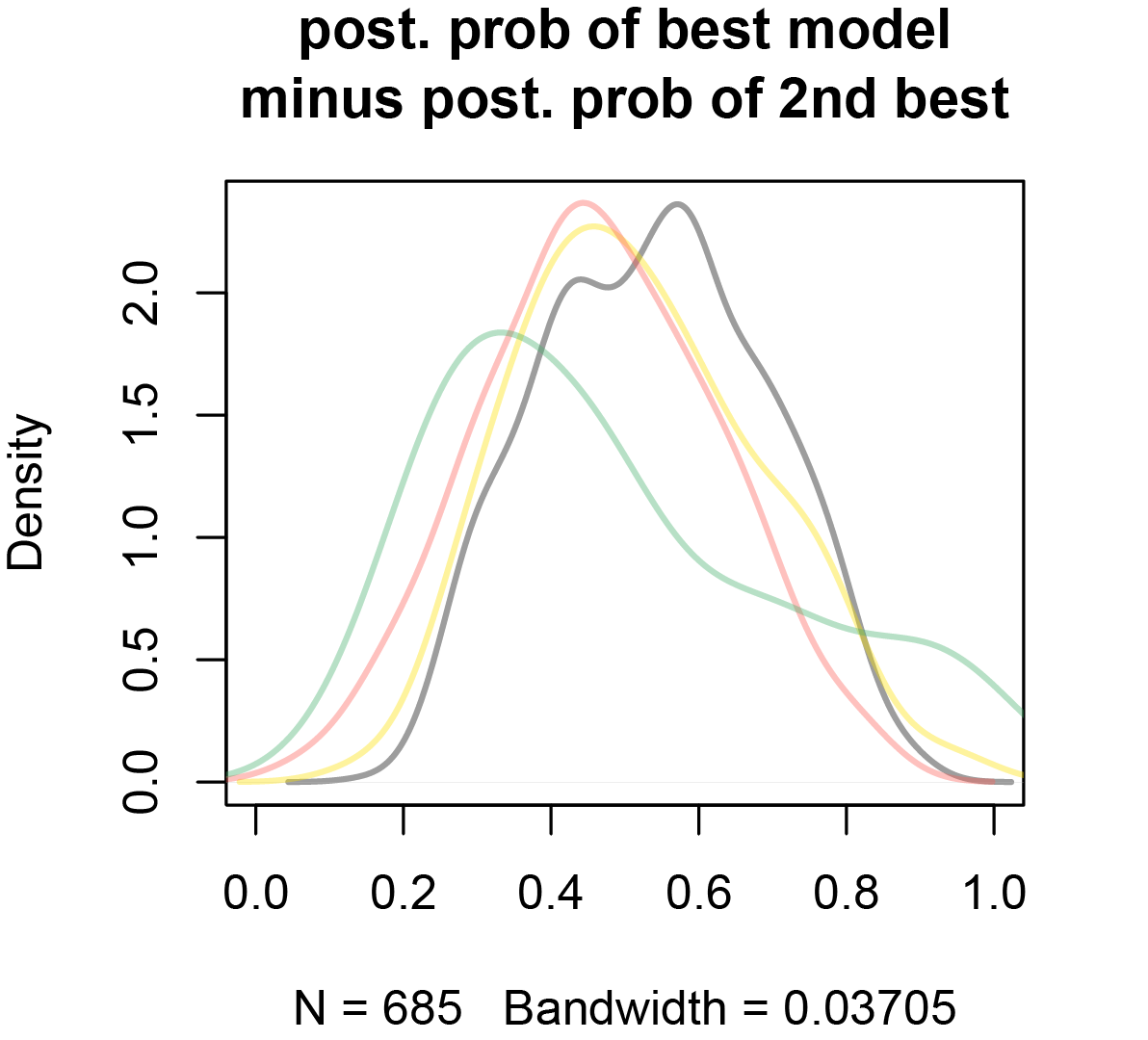

Supplement: S1 Fig — The difference between the posterior probability of the best model and the posterior probability of the second best model is plotted for genes with a posterior probability greater than 0.5 for any of the models. (TIF) [file pone.0137367.s001.tif]

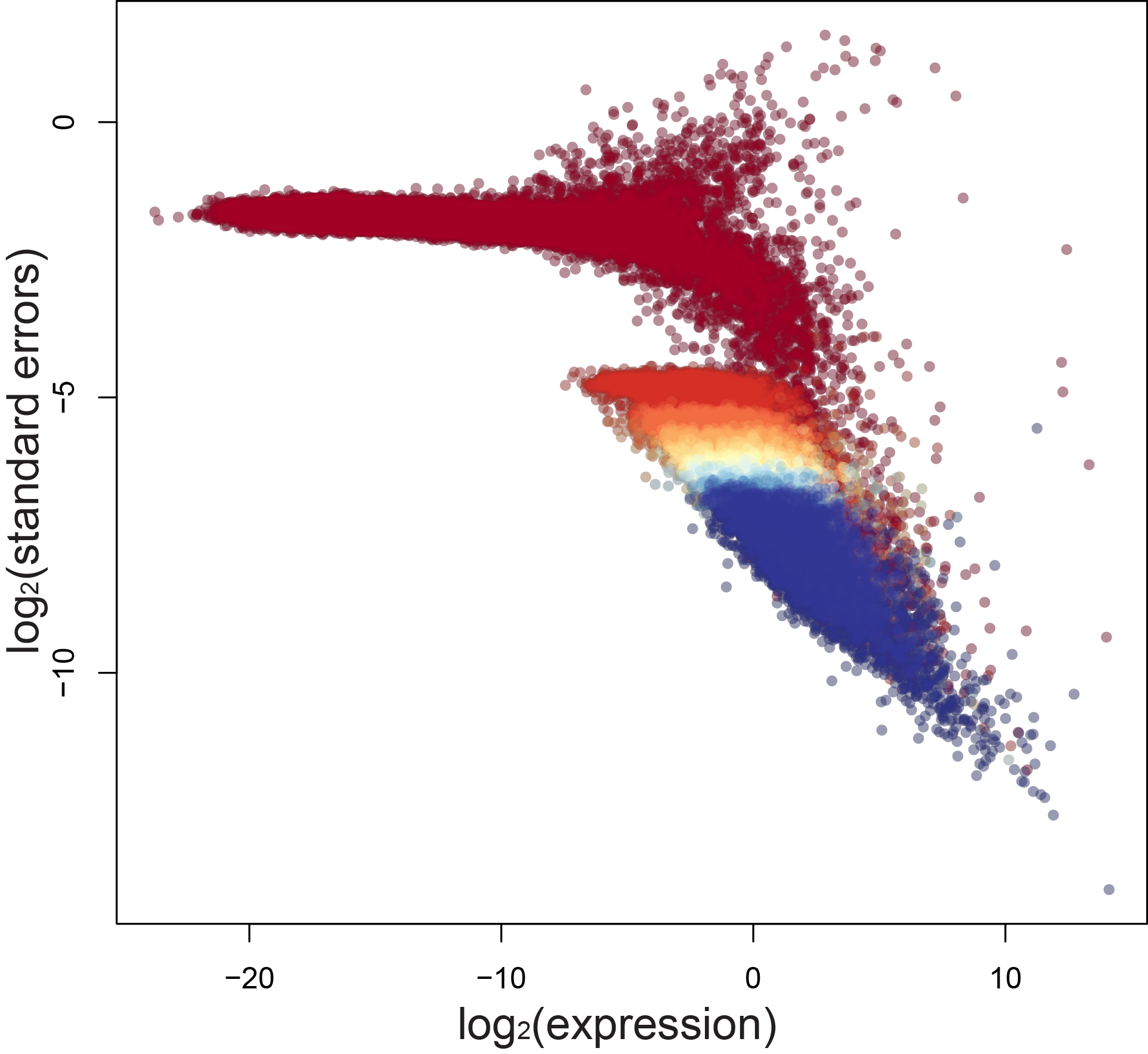

Supplement: S2 Fig — The expression levels of all transcripts in one of the F0 libraries are plotted against the respective Monte Carlo standard errors. The MCSEs are related to the expression level of the gene and to the number of reads uniquely mapping to the transcript. (TIF) [file pone.0137367.s002.tif]

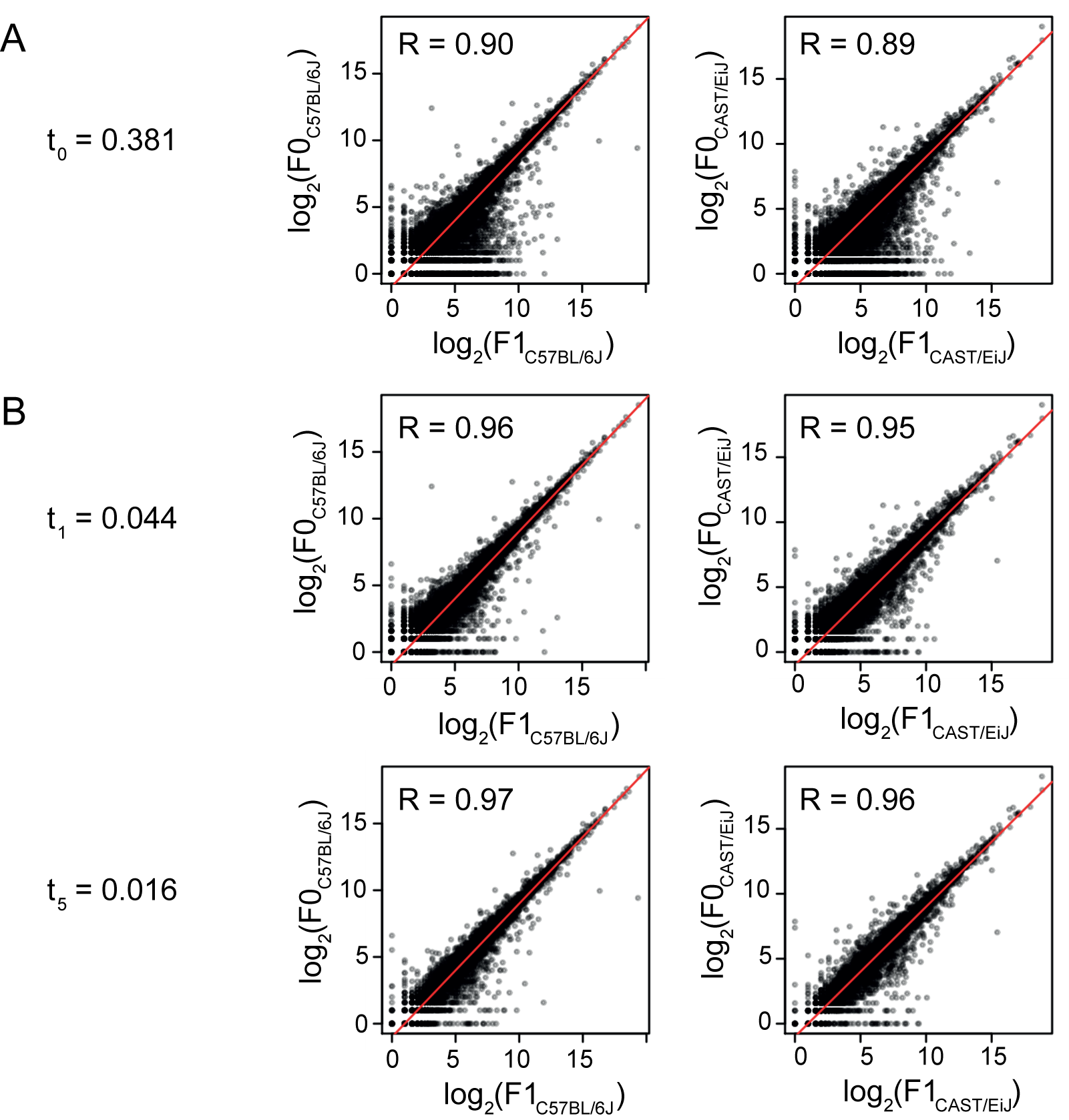

Supplement: S3 Fig — To quantify our ability to estimate allele specific isoform expression we created an artificial F1 library as described in Goncalves and Leigh-Brown et al. and compared the original expression levels to the deconvolved ones (Goncalves, Leigh-Brown et al. 2012). (A) When comparing the expression in the F0s to the allelic expression in the F1s without sub-setting by the MCSEs we found a very good agreement between the two (Pearson correlation > = 0.89). However, expression at the isoform level is less well estimated than at the gene level (Pearson correlation > = 0.97). (B) When sub setting the set of isoforms to only the ones under a MCSE threshold t (t in {t_1,t_5} corresponding to the maximum SE among isoforms with {1,5} unique reads) the agreement improves (Pearson correlation > = 0.95). (TIF) [file pone.0137367.s003.tif]

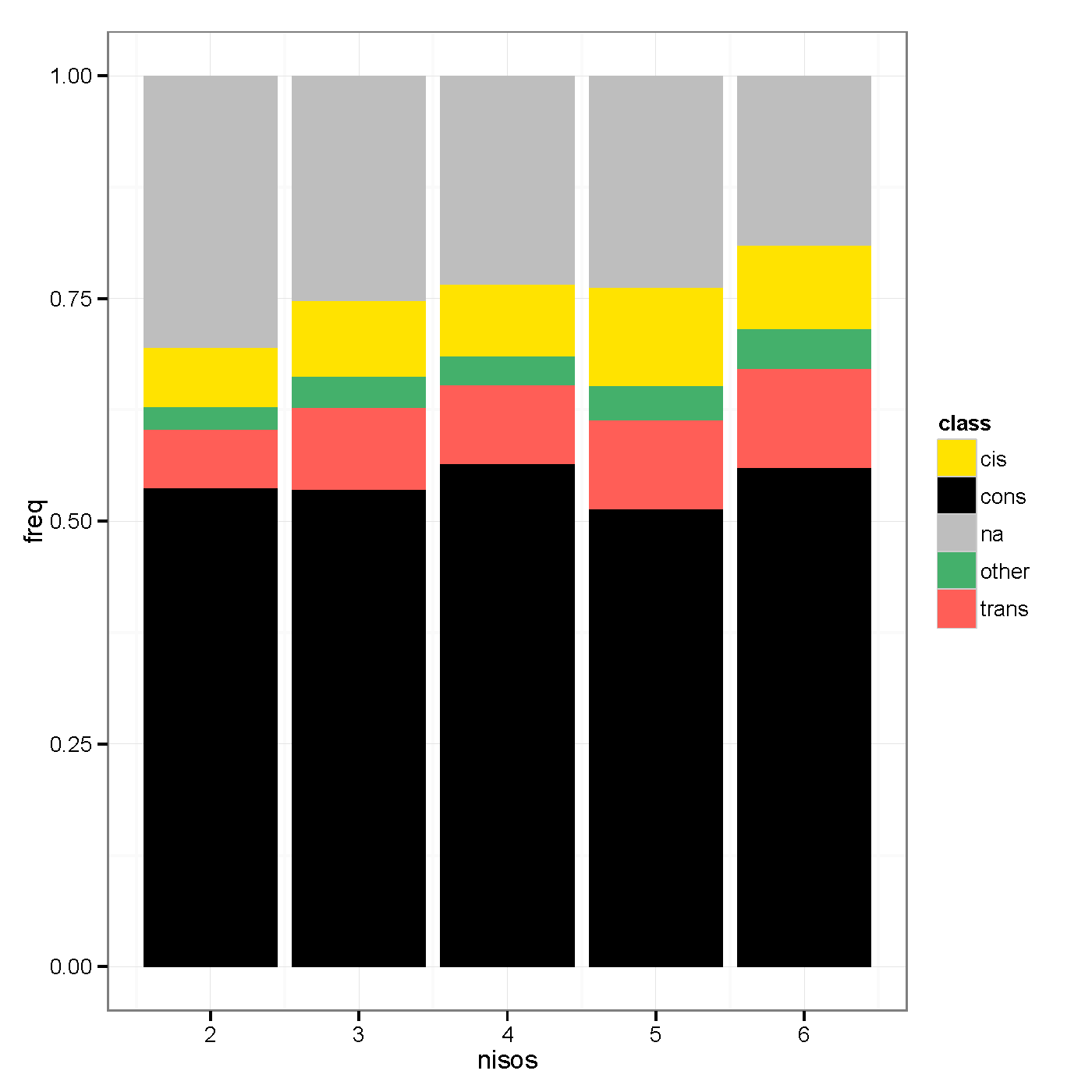

Supplement: S4 Fig — To confirm that inclusion of only genes expressing precisely 2 isoforms does not introduce a bias to the analysis, we selected the major isoform in genes expressing 3, 4, 5, or 6 (or more) isoforms and characterized them according to whether their expression in the F1 was consistent with conservation (black) or with divergence in cis (yellow), in trans (red), in cis and in trans (green). Grey indicates loci where no single model was statistically favored over the others. X-axis: number of isoforms expressed from locus, Y-axis: proportion of genes where major-isoform is most likely to have diverged due to each regulatory mechanism. (TIF) [file pone.0137367.s004.tif]
